# Nutritional Literacy for Individual Oral Health Among Village Health Volunteers in Public Health Region 1 of Thailand

**DOI:** 10.1002/hcs2.131

**Published:** 2025-02-16

**Authors:** Chollada Sorasak, Worayuth Nak‐Ai, Choosak Yuennan, Mansuang Wongsapai

**Affiliations:** ^1^ Department of Health Intercountry Centre for Oral Health Mueang Chiang Mai Thailand; ^2^ Sirindhorn College of Public Health Chonburi Praboromarajchanok Institute Mueang Chonburi Thailand; ^3^ Boromarajonani College of Nursing Praboromarajchanok Institute Mueang Chiang Mai Thailand

**Keywords:** health literacy, health region 1, individual oral health, mixed method, nutrition, nutritional literacy, oral health, oral health literacy, village health volunteers

## Abstract

**Background:**

Oral health issues persistently affect the overall health and well‐being of rural populations. Village health volunteers (VHVs) play a crucial role in advancing oral health literacy in their community. This study aimed to examine the factors related to nutritional literacy for oral health among VHVs.

**Methods:**

This was a mixed‐methods study employing an explanatory sequential design. The quantitative data were gathered through questionnaires distributed to a cohort of 10,514 VHVs registered in Health Region 1. A stratified random sampling technique was used to ensure adequate representation of various subgroups within the VHV population, considering factors such as age, education level, and geographical distribution across the region. This approach allowed for a more representative sample that accurately reflects the diversity of the VHV population. Qualitative data were obtained through semi‐structured interviews with a purposive sampling of 20 participants based on specific criteria. Quantitative data were analyzed using descriptive statistics and biserial correlation techniques, while qualitative data were analyzed using content analysis.

**Results:**

The study found that the sample group possessed a moderate level of knowledge of health literacy principles and nutrition for oral health. However, their self‐assessed skills in nutritional literacy for oral health were rated as high. A statistically significant negative correlation was found between knowledge of nutrition for oral health and skills in nutritional literacy for oral health. VHVs equated health literacy with knowledge because their public health training had focused more on imparting knowledge rather than developing skills based on health literacy principles.

**Conclusion:**

There is a need to emphasize skill‐based health literacy training and to use effective skill development techniques that are tailored to the specific roles and responsibilities of health volunteers. It is also recommended to continuously monitor and evaluate the outcomes of these efforts.

## Graphical abstract

The study examined nutritional literacy for oral health among village health volunteers (VHVs) in Thailand's Public Health Region 1. A mixed‐methods approach revealed VHVs had moderate knowledge but high self‐assessed skills in nutritional literacy for oral health, with a negative correlation between knowledge and skills. Findings highlight the need for skill‐based health literacy training tailored to VHVs' roles.

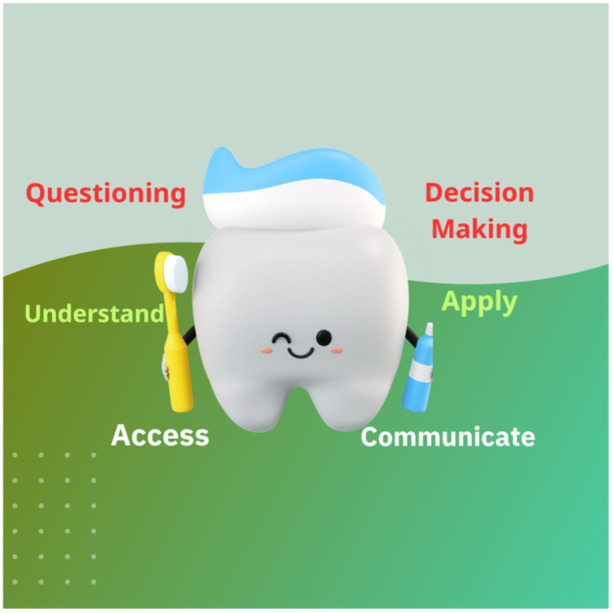

**Abbreviation**
VHV: village health volunteer

## 1 Introduction

The prevalence of oral health issues, including dental caries and tooth erosion, substantially influences tooth loss across diverse age demographics [1], with Public Health Region 1 being particularly affected. Data from the Health Data Center system, under the purview of the Ministry of Public Health, unveiled a dental check‐up rate of merely 8.80% in 2020–2022. Furthermore, only 48.22% of the population retained a minimum of 20 natural teeth and four functional posterior teeth, whether natural or artificial. The situation was markedly worse in Chiang Mai Province in 2022, where the dental check‐up rate plummeted to 5.17%, and only 62.96% of individuals possessed at least four functional posterior teeth [2]. This underscores the urgent need for comprehensive strategies to improve oral health outcomes.

Nutritional literacy is of utmost importance for oral health because inadequate knowledge can lead to unhealthy dietary behaviors that negatively impact oral health. Related studies have shown a positive correlation between good nutritional literacy and beneficial dietary practices, as well as a negative correlation with the consumption of fats and unhealthy foods [3, 4]. Individuals with low health literacy are more likely to fail to adhere to proper nutritional principles, ultimately affecting their oral health [5]. Furthermore, health literacy plays a crucial role in improving patients' quality of life [6].

The Ministry of Public Health has implemented policies to create health literacy in the general public, encompassing three key measures [7]:

(1) Driving policies and measures to create health literacy and prevent risk factors; (2) Developing public health facilities, educational institutions, and workplaces as model Health Literate Organizations; (3) Creating community‐based leaders, with village health volunteers (VHVs) playing a pivotal role as grassroots leaders in disseminating accurate health information, facilitating access to services and addressing community health issues.

The advancement of oral health literacy is an essential approach for fostering suitable health practices, mitigating disease susceptibility, and enhancing the populace's quality of life. These three initiatives work in concert to create a comprehensive framework for improving health literacy at various levels of society.

VHVs are pivotal contributors to the implementation of community public health initiatives, with support from the Ministry of Public Health. Since the inception of the 5th National Economic and Social Development Plan (1977–1981), VHVs have been an indispensable component of the national health development blueprint [8]. Their capacity to connect with the local populace and understand community concerns has contributed significantly to grassroots health promotion and disease prevention. Consequently, assessing the challenges to achieving oral health and nutritional literacy among VHVs is of paramount importance [9]. The formulation of interventions to enhance their proficiency will lead to improved oral health outcomes in rural communities.

## 2 Materials and Methods

### 2.1 Research Design

This research study was Mixed methods employed an Explanatory Sequential Design.

### 2.2 Research Methodology

#### 2.2.1 Quantitative Research

The target population for the quantitative phase comprised VHVs registered with the Division of People's Health Support in Public Health Region 1 as of October 1, 2023, totaling 135,763 individuals. Stratified random sampling was employed to ensure adequate representation of subgroups within the population. The sample was stratified on the basis of key demographic characteristics such as age, education level, and geographic location within Public Health Region 1. This approach allowed for a more representative sample that captured the diversity of the VHV population. Quantitative data were collected using a self‐administered questionnaire with a Cronbach's alpha reliability of 0.90, delivered using a Google form.

The quantitative data were analyzed using descriptive statistics, including frequency distributions and biserial correlation to establish relationships. To ensure a clear connection between the qualitative and quantitative components, we employed a content analysis approach for the qualitative data. Themes were derived through an iterative process of coding and categorizing the interview transcripts. These themes were then systematically compared and integrated with the quantitative findings to provide a comprehensive understanding of nutritional literacy among VHVs. For instance, the theme of “misconceptions about health literacy” that emerged from the qualitative data was used to interpret the quantitative results showing low scores in knowledge of health literacy principles. This integration process allowed us to explain and contextualize the quantitative findings, providing deeper insights into the VHVs' experiences and perceptions.

#### 2.2.2 Qualitative Research

The qualitative data were collected using semi‐structured interviews with 20 VHVs who had completed the quantitative survey [10]. Participants were selected using a purposive sampling approach with a criteria technique. In‐depth interviews were conducted, and the data were analyzed using analytic induction. To ensure transparency in the data integration process, we employed a systematic approach to theme extraction and quantitative‐qualitative data linkage. Qualitative data were first coded independently by two researchers using an inductive coding approach. Emerging themes were then discussed and refined through consensus meetings. These themes were subsequently mapped onto the quantitative variables using a matrix approach, where each qualitative theme was cross‐referenced with relevant quantitative measures. This process allowed us to identify areas of convergence and divergence between the two data sets. For instance, the qualitative theme of “misconceptions about health literacy” was linked to the quantitative measure of “knowledge of health literacy principles.” Discrepancies between qualitative and quantitative findings were further explored through additional analysis and team discussions to ensure a comprehensive understanding of the phenomenon under study.

### 2.3 Ethics Considerations

This research study was designed with a strong emphasis on ethical principles and the protection of participants. The study protocol was reviewed and approved by the Human Research Ethics Committee of the Sirindhorn University of Public Health, Chonburi, with Certificate of Approval (COA) number 2023/T07, obtained August 21, 2023.

## 3 Results

### 3.1 Quantitative Analysis

Part 1: Context of VHVs

1. Most VHVs (36.75%) were solely responsible for performing VHV duties, followed by 24.74% who also acted as caregivers, and 21.01% who served as local officials.
2. The sex distribution of the VHV sample shows that 82.3% were female (8651 individuals), 17.5% were male (1844 individuals), and 0.2% (19 individuals) did not specify their sex.
3. The province with the highest number of VHVs was Phayao, with 2548 individuals (24.2%), followed by Phrae with 1924 individuals (18.3%), and Chiang Rai with 1709 individuals.
4. Most VHVs (61.10%; 6425 individuals) were aged 41–59 years, followed by 28.10% (2955 individuals) aged 60 years and above, and 10.60% aged 21–40 years.
5. The vast majority of VHVs (93.5%; 9834 individuals) had an educational level below a bachelor's degree, 6.2% (654 individuals) possessed a bachelor's degree, and 0.5% (17 individuals) had a master's degree.
6. A total of 8.0% (6099 individuals) of VHVs had received training on nutritional literacy for individual oral health, while 42.0% (4415 individuals) had not.
7. When self‐assessing their overall health literacy, 55.20% (5808 individuals) of VHVs reported a high level of health literacy.

Part 2: Knowledge of health literacy principles

Table 1 summarizes the distribution of scores and incorrect responses across various health literacy skill domains. The majority (57.74%) scored 2 out of 7 points, with comprehension being the most challenging domain (97.61% incorrect responses). Decision‐making and application showed the lowest levels of misunderstanding (5.39% and 3.67% incorrect, respectively).

**Table 1:** Testing of village health volunteers' knowledge of health literacy principles (*n* = 10,514).

No.	Questionnaire item	$\bar{x}$	*SD*	Correct		Incorrect	
				*n*	%	*n*	%
1	“Knowledge” and “literacy” have the same meaning.	0.34	0.47	3539	33.66	6975	66.34
2	Health literacy means being able to read well, remember, and practice healthy behaviors.	0.18	0.38	1871	17.80	8643	82.20
3	Access is the skill of searching for diverse information and evaluating it.	0.05	0.22	543	5.16	9971	94.84
4	The skill of comprehension enables correct knowledge, understanding and beliefs.	0.02	0.15	251	2.39	10,263	97.61
5	5. The key steps in the skill of questioning/inquiry are posing questions, planning, using questions and evaluating.	0.04	0.19	385	3.66	10,129	96.34
6	The steps in the skill of decision‐making are identifying the problem, determining alternatives, evaluating and stating a position.	0.95	0.23	9947	94.61	567	5.39
7	The skill of application focuses on developing abilities, self‐reminding and self‐management.	0.96	0.19	10,128	96.33	386	3.67
	Total	2.54	0.81				

The scores were classified into five levels (Excellent, Good, Moderate, Fair, and Needs Improvement), with a range of 1.4 for each level. The overall knowledge of health literacy principles was found to be at a fair level (mean = 2.54, *SD* = 0.81), as shown in Table 1.

Part 3: Testing of knowledge of nutrition for oral health

The finding indicated that the top mode of the knowledge scores was 4 points, achieved by 2639 individuals, accounting for 25.1%. This was followed by 2 points, achieved by 2635 individuals (25.1%), and 5 points, achieved by 2129 participants (20.2%), respectively.

The scores were classified into five levels (Excellent, Good, Moderate, Fair, and Needs Improvement), with a range of 1.4 for each level. Overall knowledge of nutrition for oral health was found to be at a moderate level, with a mean of 3.60 and a standard deviation of 1.34, as shown in Table 2.

**Table 2:** Testing of knowledge of nutrition for oral health (*n* = 10,514).

No.	Questionnaire item	$\bar{x}$	*SD*	Correct		Incorrect	
				*n*	%	*n*	%
1	Adding fluoride at the correct ratio from birth to 15 years old can reduce the rate of dental caries by up to 60%.	0.14	0.35	9022	85.81	1492	14.19
2	Drinking sugar‐free or zero‐calorie carbonated beverages that use artificial sweeteners does not pose a risk of dental caries.	0.59	0.49	4324	41.13	6190	58.87
3	Gargling with salt water can help relieve toothache.	0.28	0.45	7602	72.30	2912	27.70
4	Artificial sweeteners do not cause dental caries but can actually help prevent them.	0.62	0.49	4015	38.19	6499	61.81
5	Eating fruits and vegetables cannot prevent dental caries.	0.36	0.48	6729	64.00	3785	36.00
6	Consuming highly acidic fruits can erode tooth enamel and lead to tooth erosion.	0.73	0.44	2820	26.82	7694	73.18
7	Starches and sugars are the types of foods most likely to cause dental caries.	0.89	0.32	1200	11.41	9314	88.59
	Total	3.60	1.34				

Part 4: Evaluation of nutritional literacy for individual oral health

The scores were tabulated, and the results were interpreted using a five‐level rating scale based on a five‐point Likert scale [11], with a range of 0.8 for each level. The overall evaluation of nutritional literacy for individual oral health according to the perceptions of VHVs was at a high level, with a mean of 4.06 (*SD* = 0.39), as shown in Table 3.

**Table 3:** Evaluation of nutritional literacy for individual oral health according to the perceptions of village health volunteers (*n* = 10,514).

No.	Self‐assessment topic	$\bar{x}$	*SD*	Interpretation
1	Search for additional information or knowledge from various data sources	4.13	0.56	High
2	Verify the information collected to determine what is true or false	4.01	0.59	High
3	Check the credibility of information before using or disseminating it	4.06	0.59	High
4	Check the timeliness of the information	4.01	0.59	High
5	Read and listen to information accurately and completely	4.13	0.50	High
6	Understand the information clearly	4.13	0.49	High
7	Remember the information clearly	4.07	0.53	High
8	Clearly understand the information	4.10	0.51	High
9	Prepare questions in advance	4.03	0.55	High
10	Plan the questioning	3.98	0.55	High
11	Ask medical personnel questions until doubts are resolved	4.05	0.53	High
12	Evaluate and refine the questions	4.03	0.52	High
13	Identify/specify the issues or topics requiring a decision	4.07	0.53	High
14	Determine multiple feasible options	4.08	0.51	High
15	Distinguish the pros and cons of each option	4.10	0.50	High
16	Decide and explain the rationale	4.08	0.51	High
17	Find ways to remind oneself to act, such as taking notes or setting alarms	4.04	0.57	High
18	Seek assistance from others to remind oneself	3.90	0.67	High
19	Set the goal of “drinking plain water after eating fruit” each time	4.16	0.51	High
20	Use multiple reminding methods and adjust them according to the situation	4.12	0.50	High
	Average score	4.06	0.39	High

Part 5: Relationship testing

Biserial correlation analysis [12] was used to test the relationships as described below.

1. The perceived level of nutritional literacy for individual oral health among VHVs revealed a statistically significant at the *p* = 0.01 with knowledge of health literacy principles, but the correlation was negative direction by low level. The biserial correlation analysis revealed a statistically significant negative relationship (*p* < 0.01) between VHVs' perceived level of nutritional literacy for individual oral health and their knowledge of health literacy principles. However, it is important to note that this correlation was weak and does not imply causation. The data suggest that as VHVs' self‐perceived literacy skills increased, their measured knowledge of health literacy principles decreased slightly; however, further research is needed to understand the complex relationship between these variables.
2. The perceived level of nutritional literacy for individual oral health among VHVs indicated a statistically significant at the *p* = 0.01 with knowledge of nutrition for oral health, but the correlation was negative direction by low level. This indicated that as the VHVs' perceived level of nutritional literacy for individual oral health increased, their knowledge of nutrition for oral health decreased.
3. Knowledge of nutrition for oral health showed a positive correlation with knowledge of health literacy principles at a significant level of *p* = 0.01; however, the correlation was found to be weak. This indicated that as their knowledge of nutrition for oral health increased, their knowledge of health literacy principles also increased. The results are presented in Table 4.

**Table 4:** Biserial correlation analysis.

Variable	Statistic	Knowledge of health literacy principles	Knowledge of nutrition for oral health
Skill Level Assessment of Nutrition for Individual Oral Health Literacy by Village Health Volunteers	Correlation coefficient	−0.091**	−0.073**
	*p*	< 0.001	< 0.001
	Level of relationship	Very low	Very low
Knowledge of Nutrition for Oral Health	Correlation coefficient	0.239**	
	*p*	< 0.001	
	Level of relationship	Low	

** Correlation is significant at the 0.01 level (two‐tailed).

### 3.2 Qualitative Analysis

Public Health Region 1 comprises eight provinces: Chiang Mai, Chiang Rai, Lamphun, Lampang, Phrae, Nan, Phayao, and Mae Hong Son. The study included 20 VHVs from Public Health Region 1. VHVs play a crucial role in supporting and promoting community health by providing health promotion and ongoing advice on disease treatment and disease prevention to the local population. They also serve as key change agents in terms of health behaviors by communicating public health information; providing recommendations and disseminating knowledge; planning and coordinating public health development activities; and offering various health services such as health promotion, disease surveillance and prevention, basic medical and pharmaceutical assistance according to the regulations of the Ministry of Public Health, patient referrals, rehabilitation, and community health activities. VHVs receive compensation and welfare benefits according to the regulations of the Ministry of Public Health. In this study, VHVs can be categorized into two main groups: (1) VHVs who exclusively perform their duties as village health volunteers, and (2) VHVs who serve both as village health volunteers and as committee members or participants in other community organizations. Examples include caregivers for the bedridden elderly, members of women's development groups, savings groups, volunteer teachers, or members of local administrative organizations. Although most VHVs have attained an educational level under a bachelor's degree, they demonstrate significant potential and capability in community development through collaboration and support from both public and private sectors. The analysis revealed two main themes: understanding of oral health nutrition literacy and experiences related to oral health nutrition literacy.

Theme 1 *Understanding of oral health nutrition literacy*

The participants demonstrated diverse interpretations regarding the distinction between knowledge and health literacy. One portion of the participants interpreted both concepts as identical, articulating for example, “There are the same, right?” (P1). Conversely, some participants expressed uncertainty regarding the distinction between them, saying, “They're not the same, but I don't understand. Nothing has changed, even after the training and meetings, it's still the same as before” (P13).

Regarding skill in accessing information, participants expressed different perspectives. Some reported receiving information from various media “I got from various media channels” (P7, P11, P13, and P20), “I got it from dentists, they were providing knowledge and from hospital public relations” (P1 and P11), while several participants had the ability to search for information on their own “I can search for it on Google” (P1, P3, P4, P6, P9 and P13).

Participants reported possessing an understanding of the communication process, including the elements of a communication model (sender, message, channel and receiver), but their understanding was not comprehensive. As one participant stated, “The content is sometimes similar, but I don't know if it's old or new. The content is from what year, it's hard to find” (P10 and P16).

Many participants understood that the ability to comprehend information or messages would be improved if the content or text was short and clear, easy to remember and accompanied by visuals; for example, “There needs to be videos or pictures, otherwise, I won't understand” (P1, P7, P8, P9, P10, P12, P13, P15, P17, P18, and P20).

Although the participants demonstrated an understanding of the concept of inquiry, most struggled to formulate clear and goal‐oriented questions to obtain the desired information. Even those who claimed to be capable of asking questions did not evaluate their inquiries afterward. For instance, one participant stated, “I can't ask questions, but I'll just repeat what the doctor said before; otherwise, they'll think I don't know” (P3 and P10). Another participant mentioned, “I didn't prepare any questions. If I think of something when I meet the doctor, I'll just ask” (P2 and P8).

In relation to the awareness that consumption of fizzy drinks can lead to tooth decay, participants exhibited diverse decision‐making approaches. Some opted to cease drinking such beverages altogether and transitioned to plain water “Decided to stop drinking fizzy drink and then switch to drinking plain water” (P9). Conversely, others persisted in their soft drink consumption, rationalizing their behavior with sentiments like: “Decided to still drink, I already love to drink. If I have decayed teeth, I'll see the dentist” (P18). Others decided to reduce their consumption without considering it harmful “Decided to drink less. Anyway, I don't drink much in the first place” (P20).

Participants understood the crucial need to practice what they learned. Factors contributing to effective application included the ability to remember it themselves “Remember in my mind to eat fruit and then drink water” (P13), and the ability to remind themselves, as well as seeking support from others or using reminder tools “Set a timer on my phone to drink water after eating fruit” (P8), “Ask relatives or close people to help remind me” (P10 and P11), and “Write it on the water bottle” (P9).

Theme 2 *Experiences related to oral health nutrition literacy*

In the context of health literacy, VHVs were unable to explain the different between knowledge and literacy even after attending the training sessions, meetings, and seminars, which adhered to conventional teaching methods without significant modifications or advancements. Notably, VHVs did not receive explicit training focused on health literacy skills. As articulated by one participant, “Teaching and training techniques for health literacy among public health personnel still rely on traditional lecture‐based methods rather than fostering learning processes that develop health literacy skills.” (P1). Similarly, another participant recounted, “During VHV meetings, the doctor's presentation included slides that referenced the term ‘literacy’ ” (P2, P4, P5, and P8). Ultimately, despite training and ongoing meetings, the status quo persisted, as expressed by P13, “Nothing has changed; it remains as it was before.”

Regarding access to information, most VHVs had experiences of self‐searching “Searched from Google” (P1, P3, P4, P6, P9, and P13), “In the past, it was newspapers, now I read books about teeth and oral cavity or ask the doctor. If I don't understand, I will consult dental expertise” (P6 and P16), and “These days, I use Facebook, Line, there's information to read, YouTube/TikTok too” (P4 and P13). Some also received information directly from experts through training “Received training from expert staff who came to teach in the village” (P15 and P17).

In the context of communication, VHVs possessed experience in disseminating health information to the local population; however, they frequently encountered challenges. The VHVs occasionally lacked credibility and preparedness, leading recipients to harbor skepticism toward the information provided as they mention “The people we teach don't believe in the importance of teeth; they say we're not dentists” (P1 and P5). VHVs also sometimes lacked understanding and the ability to identify the main points in the information to be communicated and failed to verify the timeliness of the information “Just forwarded the message to the target group, sometimes I don't really understand but I just send it, through Line or give out brochures, sometimes I even gave out outdated brochures” (P3 and P9), and “It was like that before, where I couldn't explain further, like the 222 toothbrushes, when people in the area asked, I couldn't answer” (P14, and P19). Additionally, confusion existed between the message and the communication channel, and recipients' adherence to existing beliefs hindered effective communication “They don't really believe the VHVs; they believe that if their teeth hurt, they should just go see the doctor” (P17 and P19).

Regarding comprehension, VHVs were unable to remember and grasp the key points of complex oral health information, which could impact their ability to provide proper care and practice oral health. This was often due to the complexity of the oral health information, which is full of medical terminology and difficult for the general public to understand. The presentation of the information also did not facilitate learning and lacked appropriate formatting, visuals, or media to aid in remembering and understanding. As one participant stated, “Some dental issues are hard to understand, a lot of English words need to be put in Thai” (P1, P2, P11, P14, P15, P16, P19, and P20). Lack of interest and awareness of the recipients also affected their ability to understand and remember the information “The patients don't really care about what we tell them, when we visit again, they can't remember what we said” (P6 and P12). The abundance of information in the digital age also made it difficult to filter and remember the useful information “There's too much information about good foods for teeth online; I get confused about which one is correct” (P14 and P19). Many participants also lacked regular reviews or opportunities to practice teaching the information received, which could further impede their ability to remember oral health information “If you only teach once, I can't remember some items; if I don't try to practice, I still won't understand. It needs to be taught many times before I can remember and understand” (P1, P2, P3, P5, P7, P8, P10, P11, P12, P14, P15, P17, P18, P19, and P20). Personal beliefs and cultural values can also lead to different understandings or perceptions of oral health information “Some people believe that sucking on salt can relieve toothache; they won't go see the doctor, no matter what you tell them, they won't believe you” (P10 and P15).

In terms of inquiry skills, VHVs gained practical experience in eliciting medical histories from patients within the community. However, their use of questioning techniques often lacked strategic planning and subsequent evaluation “I didn't prepare any questions. If I think of something when I meet the doctor, I'll just ask” (P2 and P8) and “I asked, but I didn't think about the question further, as long as I got the answer I wanted” (P6 and P17).

In terms of decision‐making, VHVs demonstrated two types of decision‐making skills: (1) evaluating alternatives and (2) weighing pros and cons “Decided not to drink, because drinking causes stomach acidity” (P2), “Decided not to drink; it's very inappropriate to drink because it's an acid that destroys health. And from what I've heard and experienced before, they use it to clean engines, remove rust, clean bathroom stains, and other items, so for personal health, it's best not to drink it at all” (P7), and “Decided to stop drinking sugary drinks because they cause tooth decay” (P8, P14 and P17). Some VHVs made decisions without benefit evaluating by comparing other alternatives choices “Decided based on the situation and context of each case” (P19) and “Decided to change behavior by reducing the frequency of drinking” (P12).

Regarding application, VHVs had plans to remind themselves, ask others to help them, or use reminder tools “Use the method of remembering and practicing until it becomes a habit” (P1), “Set a reminder note on my mobile phone” (P12), “Asked relatives or close people to help remind me” (P10, P11), and “Shared information in the Line group” (P17).

### 3.3 Data Integration

**Case 1:** Quantitative study data revealed that the correlation analysis showed when the score of nutrition for individual oral health literacy increases, it results in a decrease in the score of knowledge of health literacy principles and also leads to a decrease in the score of knowledge of nutrition for oral health.

Although 58% of the VHVs have received training in nutrition for individual oral health literacy and self‐assessed their knowledge of health literacy and nutrition for individual oral health literacy, but the qualitative study results indicated that VHVs still lack the skills and knowledge to apply the health literacy principles in their work.

This finding was consistent with the VHVs' understanding that no difference existed between knowledge and literacy. Their training followed the same traditional teaching methods without any changes or development, and they did not receive clear training on health literacy skills.

Regarding communication, while VHVs communicated with the community, they often encountered problems, indicating a lack of communication skills. They also struggled to remember and comprehend complex oral health information because the information often contains medical terminology and is presented in a way that does not facilitate learning, lacking appropriate formatting, visuals, or media.

**Case 2:** The quantitative analysis showed that the VHVs' knowledge of nutrition for oral health increased, and their knowledge of health literacy principles also improved. This finding was consistent with the qualitative findings that VHVs often sought help from dentists to understand oral health‐related issues. Training and information for VHVs were provided by dentists and hospital public relations staff. Additionally, 58% of VHVs had received training in nutritional literacy for individual oral health, which explains the increase in knowledge of health literacy principles.

## 4 Discussion

1. Most VHVs were female and worked solely as VHVs, with most aged 41–59 years with an educational level below a bachelor's degree. However, they assessed themselves as having a high level of knowledge in the areas of oral nutrition and personal health literacy. The predominance of women as VHVs reflects the significant participation of women in community‐level public health work, consistent with research showing that women tend to have higher ethical standards, which is an important factor in the successful implementation of community health initiatives [13]. The study found that community empowerment continues to be a crucial factor in enhancing women's participation [14]. The largest age group of VHVs was 41–59 years (61.10%), followed by those aged 60 years and older (28.10%), indicating that most VHVs are of working age or retirees, suggesting that they have the time and commitment to support health activities in the community. Because they typically engage in independent occupations or agricultural activities on their own land situated within the same community. This finding aligns with studies showing that women living in the community for a long time and having free time from work are more likely to participate in volunteer activities, with the main barrier for nonparticipation being a lack of time to engage in volunteer work [15]. It also corresponds with the suggestion that activities should be organized to promote good health among the elderly and facilitate their social participation, especially through religious activities, to help older adults in retirement feel valued and adapt effectively [16].
  Regarding education, 93.5% of VHVs possessed an educational level below bachelor's degree, suggesting that a basic level of education may be sufficient for this role. However, the researchers suggest that training programs should be considered to enhance the VHVs' specific knowledge and skills. More than half of VHVs (58.0%) received training in nutritional literacy for individual oral health, indicating that efforts to improve the knowledge and capabilities of VHVs in health literacy enhanced their effectiveness in promoting community health. Additionally, 55.20% of VHVs assessed themselves as having a high level of health literacy, which is a positive sign of their confidence in their own capability to perform their duties. However, the researchers believe that this study also reflects the VHVs' need for social acceptance, even with an educational level below bachelor's degree, aligning with Maslow's concept of the need for esteem and recognition from close individuals, other groups, organizations, and teams [17]. The researchers concluded that additional training opportunities should be provided to enhance the knowledge and skills necessary for sustainable public health support at the community level.
2. The results indicated that more than 60% of VHVs lack fundamental knowledge of health literacy principles, which included skills in understanding, inquiry, accessibility, the definition of health literacy, and the distinction between “knowledge” and “literacy”.
  Additionally, their knowledge of nutrition for oral health was higher than their knowledge of health literacy principles. The VHVs' self‐assessment of their nutritional literacy for individual oral health was at a high level, reflecting a lack of understanding of health literacy principles. Qualitative analysis suggests that the VHVs equate literacy with knowledge as a result of their experiences in public health training, which focused on providing knowledge rather than skill‐building in line with health literacy principles.
  To address this issue, the researchers propose a revision of the training program, emphasizing both knowledge enhancement and skill development based on the components of health literacy, in alignment with the principles outlined by Fitts and Davies [11, 12]. This approach entails deconstructing complex skills into smaller components, systematically practicing each element, and progressively honing proficiency through iterative repetition and constructive feedback, a strategy known to accelerate skill acquisition and enhance effectiveness [18, 19]. The curriculum development process must align with the specific issues and needs of individuals, corresponding to the educational requirements of Azza and Susilo [20], which suggest that female students in boarding schools need reproductive health education to cultivate healthy living habits. Training schedules should be codified as strategic health policy components, aligned with Desfiani et al.'s [21] insights advocating capacity‐building programs enhancing information search skills, positioned as a strategic imperative to address COVID‐19 challenges.
  The findings of this study also reflect the level of knowledge of VHVs regarding health literacy principles and nutrition for oral health. It was found that their basic knowledge of health literacy principles was at an acceptable level, while their knowledge of nutrition for oral health was at an average level, indicating a certain level of understanding but with room for improvement and development. This highlights the need to enhance the effectiveness of training through practical exercises, aligning with Dewey's [22] concept that education should emphasize learning from experience through training that focuses on skill development and real‐world practice in authentic situations, fostering understanding and proficiency in learners' day‐to‐day work. This resonates with Nadler's concept of capacity development, emphasizing learning about one's current job, aiming to develop knowledge, abilities, and skills that enable trainees to immediately apply their acquired knowledge to their work [23].
  The VHVs' self‐assessed their nutritional literacy for individual oral health at a high level. This may reflect their perceived importance and confidence in applying this knowledge in their work as VHVs, in line with Bandura's concept that the more individuals perceive their capabilities and inherent potential, the more they can effectively use their abilities in their work or daily lives to their full potential. Those who perceive their abilities more accurately tend to achieve more [24].
  However, on the basis of these findings, the researchers believe that the training program should be reviewed and improved to ensure a thorough understanding of the core principles of health literacy and appropriate skill development in nutritional literacy for individual oral health. This will facilitate the effective application of these principles in the VHVs' duties, promoting sustainable community health.
3. The study findings reveal that VHVs equate the terms “literacy” and “knowledge” as a result of their experiences in public health training, which emphasized the provision of knowledge rather than the development of skills according to health literacy principles. This is a significant problem that needs to be addressed because health literacy involves the perception of health information and the ability to correctly analyze and synthesize such information. Therefore, training VHVs in the skills of understanding and communicating health‐related matters and decision‐making is crucial.
  The researchers also underscored the importance of dispelling the misconception that health literacy is synonymous with mere factual knowledge. The World Health Organization defines health literacy as “the degree to which individuals possess the capacity to acquire, process, and comprehend fundamental health information and services necessary for making informed health‐related decisions” [25]. Consequently, training programs should extend beyond knowledge dissemination to cultivate essential health literacy competencies. These include the ability to access, comprehend, inquire, decide, and effectively communicate a multifaceted skill set indispensable for VHVs to proficiently navigate their daily healthcare responsibilities. Improvements in staff knowledge and behaviors after health literacy educational interventions should be highlighted [26, 27, 28]. Despite an emphasis on all health literacy techniques during this training, the reported use of plain language showed no improvement post‐training [29].
  To address this issue, the researchers suggest that public health training courses should focus on developing both knowledge and skills based on the components of health literacy. Courses should be tailored to the specific context and the roles of the learners. In the case of VHVs, the necessary skills include accessing and understanding information, inquiring, decision‐making, applying, and communicating, which should be systematically taught and practiced to ensure effective application in their daily healthcare activities and to promote sustainable community health.
  However, the study faced limitations in terms of financial resources, which restricted the ability to access all the target interview participants across different provinces. Challenges in establishing partnerships with supporting organizations or agencies for research and education also posed difficulties in overcoming the financial constraints.

## 5 Conclusion

Despite the VHVs' self‐assessment of having a high level of nutritional literacy for individual oral health, the study found that more than 60% lacked a fundamental understanding of health literacy principles, including skills in comprehension, inquiry, access, the meaning of health literacy, and the distinction between knowledge and literacy. This finding highlights the need for comprehensive training programs that focus on both providing knowledge and developing skills based on the components of health literacy principles, by adopting training approaches such as those proposed by Fitts and Davies [11, 12], involving breaking down complex skills into smaller components and building proficiency through repetition and feedback.

The study also revealed the importance of addressing the misconception that health literacy is synonymous with knowledge, which is contrary to the World Health Organization's definition of health literacy as the ability to access, understand, and use health information to make appropriate health decisions. Therefore, the training programs for VHVs should provide knowledge and cultivate the necessary health literacy skills, such as accessing and understanding information, inquiring, decision‐making, and communicating, to enable them to effectively apply health information and make informed decisions, ultimately enhancing the efficiency and sustainability of community‐level health promotion.

## Author Contributions

**Chollada Sorasak:** conceptualization (equal), data curation (equal), formal analysis (equal), investigation (equal), methodology (equal), project administration (equal), resources (equal), software (equal), supervision (equal), validation (equal), writing–original draft (equal), writing–review and editing (equal). **Worayuth Nak‐Ai:** visualization (supporting), methodology (equal), supervision (equal), validation (equal), original draft (equal), writing–review and editing (supporting). **Choosak Yuennan:** data curation (supporting), formal analysis (supporting). **Mansuang Wongsapai:** resources (supporting), supervision (supporting), validation (supporting).

## Ethics Statement

The study protocol was reviewed and approved by the Human Research Ethics Committee of the Sirindhorn University of Public Health, Chonburi, with Certificate of Approval (COA) number 2023/T07, obtained August 21, 2023.

## Consent

All research participants provided written informed consent at the time of entering this study.

## Conflicts of Interest

The authors declare no conflicts of interest.

## Data Availability

The authors have nothing to report.
